# Testing the Validity of Self-Reported Posttraumatic Growth in Young Adult Cancer Survivors

**DOI:** 10.3390/bs8120116

**Published:** 2018-12-15

**Authors:** Crystal L. Park, Sinead M. Sinnott

**Affiliations:** Department of Psychological Sciences, University of Connecticut, Storrs, CT 06269, USA

**Keywords:** posttraumatic growth, psycho-oncology, adolescent and young adult cancer survivors, wellbeing

## Abstract

Posttraumatic growth has garnered increasing interest as a potential positive consequence of traumatic events and illnesses. However, scientific investigations have yet to demonstrate the validity of self-reports of posttraumatic growth. The most common measure used to assess this construct is the Post Traumatic Growth Inventory (PTGI); however, the extent to which the PTGI (as well as other self-report measures of perceived posttraumatic growth; PPTG) assess actual positive change remains unknown. The present study aimed to examine the validity of PPTG measures. We assessed 83 adolescent and young adult (AYA) cancer survivors at two time points, one year apart. We measured the stability of PTGI from T1 to T2, correlated three measures of PPTG that used different methods (only positive, positive or negative, positive and negative change) with wellbeing measures, and compared PTGI scores with changes in psychosocial resources. PTGI scores were stable over time. More nuanced measures of PPTG appeared to capture more perceived change, although no measure of PPTG was favorably related to wellbeing. Finally, PTGI did not correlate with change in psychosocial resources, with the exception of spirituality. Overall, our results suggest that measures of PPTG do not capture actual positive changes experienced by AYA cancer survivors.

## 1. Introduction

The ability to learn and grow from difficult or challenging life experiences is a key aspect of individual development and human survival. Scholars have been interested in this idea for many years [1], and empirical studies began to surge in the late 1990s, a pace that continues unabated. Clearly, this notion has strong intuitive appeal and has seen not only increasing scholarly interest but also attention by the lay public [2].

Unfortunately, methodological approaches to studying growth following adversity have not kept pace with clinical and popular interest. In particular, virtually all of the research that has attempted to assess posttraumatic growth (PTG) relies on self-report measures of this construct [3,4], even though the accuracy of these reports has been strongly critiqued [5]. In spite of the hundreds of empirical studies using measures such as the Posttraumatic Growth Inventory (PTGI) [6], very little research has demonstrated the validity of these self-report measures as actually assessing change. The few studies that have directly compared perceived PTG (PPTG) and observed PTG demonstrated that these phenomena are distinct and essentially unrelated [5]. In the present study, we aimed to contribute to this important topic a consideration of the extent to which PTG is reflected in reports of PPTG.

Since much—perhaps most—of the research on PPTG has been conducted in the context of cancer, given the highly stressful circumstances and long posttraumatic period that many survivors experience [7], we examined issues of validity of PPTG in this group. In particular, we focused on adolescent and young adults (AYAs), who are at an important developmental point in their lives, who likely will have a long period of survivorship, and for whom cancer was clearly an off-time event [8]. We aimed to test the validity of PPTG as a result of having had cancer three different ways: (1) stability, (2) relations with wellbeing and extent to which assessment method can affect relations with wellbeing, and (3) relations with directly-measured psychosocial resources. We reasoned that:

(1) If valid, PPTG should increase over time, particularly in a group with high normative developmental tasks as they head further into survivorship and are provided more opportunities to experience positive changes. Especially in AYA survivors, personal growth should increasingly manifest as individuals pursue their adult social, career, educational, and family roles. Thus, if PPTG reflects accurate reports of growth, we would anticipate higher levels of growth over time. On the other hand, stability in PPTG would suggest that PPTG reflects a trait-like variable. Relatively few studies have examined the stability of PPTG, and those that have generally have found impressive stability over time, even for periods up to eight years [9]. A recent study of AYAs similar to the present sample found reports of PPTG stable in over two-thirds of the sample across 1.5 years [10]. 

(2) If valid, PPTG should be associated with greater wellbeing. If PPTG accurately reflects positive changes, we would expect it to favorably relate to wellbeing. However, the literature is very inconsistent in this regard: some studies have found that PPTG predicts better adjustment [11], others that it predicts worse [12], and still others find no relationship at all [13]. Studies showing positive relations between PPTG and wellbeing often fail to control for potential confounding variables (e.g., mastery, optimism). In AYA samples such as that studied here, PPTG has been associated with poorer mental and psychological wellbeing and unrelated to quality of life [8]. 

The lack of consistent findings across samples may be due to methodological issues, particularly measurement. Instruments like the PTGI inherently convey to respondents that high levels of growth are possible, potentially biasing them to over-report growth. Furthermore, many of the most commonly-used instruments (e.g., PTGI, Stress-Related Growth Scale; SRGS [14]) do not provide individuals the option to report having experienced negative impacts from events, only positive ones. Researchers have found that much higher levels of PPTG are reported when participants are administered such single-valenced instruments, compared to levels of PPTG on scales providing options for reporting change ranging from highly negative to highly positive [15]. Furthermore, in studies in which individuals were asked to rate change or impact using a range from negative to positive, relationships of PPTG with wellbeing appear much less robust, especially relative to the fairly robust inverse relationships of perceived negative changes with wellbeing [15,16,17]. 

If reports of growth are accurate, we would expect PPTG to be positively associated with wellbeing and changes in PPTG over time to be positively associated with changes in wellbeing over time. Furthermore, comparing association of PPTG assessed as solely positive change, as positive or negative change, and as changes that are separately positive and negative with multiple aspects of wellbeing may illuminate the effect of different methods of assessing PPTG. We would expect, if PPTG is valid, that these methods would draw for, respectively, relatively greater, lesser, and moderate amounts of over-reporting bias, which would be reflected in relatively weaker, stronger, and intermediate strength of favorable relationships with wellbeing. 

(3) If valid, PPTG should be associated with higher levels of psychosocial resources. PPTG has been associated with psychosocial resources (e.g., optimism, social support, spirituality) in many studies [18], including in AYAs [19], and may be interpreted as providing support for validity. However, most of these studies conceptualized psychosocial resources as precursors of growth rather than as concomitant reflections of positive changes. The handful of studies that examined resources as evidence of validity have demonstrated tepid support. For example, longitudinal studies of undergraduates reported modest correspondence between PPTG and increased levels of some relevant resources (e.g., spirituality, optimism) but not others (e.g., family relationships) [12,20]. 

## 2. Materials and Methods

### 2.1. Recruitment

This analysis uses data collected for a larger study examining AYA wellbeing, funded by the LIVESTRONG Foundation. Participants were recruited by mailing materials to AYA survivors identified through the Hartford Hospital Cancer Registry. In addition, some participants were identified through AYA survivor websites, using links that directed interested participants to an online version of the survey. Participants include individuals diagnosed with cancer when they were between 15 and 39 years old and within seven years of study recruitment and who self-identify as survivors. Each participant was assessed twice, one year apart. This study was approved by Institutional Review Boards at Hartford Hospital and the University of Connecticut.

The final sample included one hundred twenty participants at Time 1 (T1) along with 13 participants excluded because of missing data (see Table 1). T1 included 88 women, 25 men, and 7 individuals who did not report gender. One year later, 66.2% of participants (*n* = 83) completed the follow up study at Time 2 (T2). Nine of the 92 participants who partially completed T2 were excluded because of missing data. At T1, participants’ ages ranged from 16 to 47 (M = 33). Participants’ age at diagnosis ranged from 15 to 39 years (M = 29). Mean time since diagnosis was 3.83 years.

### 2.2. Measures

#### 2.2.1. Perceived Posttraumatic Growth Measures

We measured PPTG comprehensively using three scales. To examine validity, we focused primarily on the measure most commonly used to assess PPTG (the PTGI). Additionally, we included a measure that examined perceived impact of cancer (ranging from highly negative to neutral to highly positive) on a variety of domains specifically relevant to AYAs (the Late Adolescence and Young Adulthood Survivorship Related Quality of Life Scale; LAYA-SRQL) [21], and a measure that separately assessed perceived change in eight cancer-relevant domains: four regarded as positive and four regarded as negative (the Impact of Cancer Scale; IOC) [22]. We included a broad range of cancer-relevant wellbeing measures, given that some researchers have suggested that PPTG might be more strongly associated with positive aspects of wellbeing (e.g., life satisfaction) as opposed to negative ones (e.g., depressive symptoms) [23] and with specific aspects of those dimensions (e.g., anxiety rather than depression) [24].

The Posttraumatic Growth Inventory-Short Form (PTGI) is a 10-item measure that assesses positive outcomes after a potentially traumatic event. It includes five subscales: new possibilities (e.g., “I established a new path for my life”), relating to others (e.g., “I have a greater sense of closeness with others”), spiritual change (e.g., “I have a better understanding of spiritual matters”), personal strength (e.g., “I know better that I can handle difficulties”), and appreciation of life (e.g., “I have a greater appreciation for the value of my own life”). Items are rated on a scale from 0 (did not experience) to 5 (very great degree). Previous studies have shown that results on the short form are similar to those of the full scale [25]. The scale had strong internal consistency reliability in our sample (*a* = 0.87).

The Impact of Cancer Scale- version 2 (IOC) measures long-term quality of life and wellbeing after cancer. It consists of four positively-valenced subscales (empathy, health/self-care, meaning, and positive self-evaluation; e.g., “I feel a sense of pride or accomplishment from surviving cancer”), and four negatively-valenced subscales (health concerns, body changes, life interference, and cancer worry; e.g., “Having had cancer makes me feel unsure about the future”). Items are rated on a five-point Likert scale from 1 (strongly disagree) to 5 (strongly agree). The scale has been shown to have good internal consistency and construct validity [22,26]. Within our sample, the negative subscales had excellent (*a* = 0.81–0.92), and the positive subscale had good internal consistency reliability (*a* = 0.75–0.86).

The Late Adolescence and Young Adulthood- Survivorship Related Quality of Life measure (LAYA-SRQL) is a scale that assesses long-term adjustment and life satisfaction in domains especially relevant to the AYA survivor population. It includes the same 30 items assessing the satisfaction and then the impact of life domains. For the purposes of our study, we used only the impact scale responses. Impact scale instructions read, “For each item, please indicate whether you have changed in this way as a result of your experience with cancer in either a positive or a negative direction. If the item is not relevant to you, please mark ‘not applicable.’”. Responses spanned a 7-point Likert scale from 1 (very negative impact) to 7 (very positive impact), or 0 (not applicable). Responses for each impact item were transformed into separate positive (5 = 1, 6 = 2, 7 = 3, 1–4 = 0) and negative (3 = 1, 2 = 2, 1 = 3, 4–7 = 0) impact scores. Example subscales include “Spirituality”, “Intimacy”, and “Family”. The scale has been shown to have good validity and reliability [21]. Our sample showed excellent internal consistency reliability for both the positive/growth subscales (*a* = 0.73–0.94) and the negative/decline subscales (*a* = 0.76–0.96).

#### 2.2.2. Wellbeing Measures

Our wellbeing measures included measures of distress as well as general affect and life satisfaction. 

The Depression Anxiety Stress Scale- 21 Item (DASS-21) is a 21-item scale, measuring how often the participant experienced symptoms of depression (e.g., “I felt I wasn’t worth much as a person”), anxiety (e.g., “I felt I was close to panic”), and stress (e.g., “I found it hard to wind down”) in the past week. Each item is scored from 0 (never) to 3 (always). We used a combined score of the three domains. It has demonstrated good validity in non-clinical samples [27]. Within our sample, the scale showed strong internal consistency reliability (*a* = 0.89).

The Impact of Events Scale-Revised (IES-R) is a 22-item instrument that assesses three domains of posttraumatic stress symptoms including intrusions (e.g., “Pictures about it popped into my mind”), hyperarousal (e.g., “I was jumpy and easily startled”), and avoidance (e.g., “I stayed away from reminders about it”). Participants responded with frequency with which they experienced each symptom in the past week with regard to their cancer, on a scale of 0 (not at all) to 4 (extremely) summed for a total score. It has shown good construct validity and internal consistency [28]. Within our sample, the scale demonstrated excellent internal consistency reliability (*a* = 0.91). 

The Concerns about Recurrence Scale CARS assesses the frequency and consistency of worries related to cancer returning with eight items (e.g., “How much time do you spend thinking about the possibility that your cancer could recur?”) rated from 1 (not at all) to 6 (very much). It has been shown to be internally consistent and valid [29]. Internal consistency reliability for the present sample was good (*a* = 0.75).

Quality of Life Inventory (QLI) is a commonly used quality of life measure used to assess spiritual (e.g., “Your faith in God”), family (e.g., “Your ability to take care of family responsibilities”), social (e.g., “The emotional support you get from people other than your family”), and health (e.g., “The amount of pain that you have”) domains of wellbeing. It presents the same 33 items twice, first inquiring about satisfaction with the items, then personal importance of the items. Scores are weighted by multiplying the importance of the item by satisfaction with it. The scale has good reliability and validity [30]. Within our sample, the subscales showed good reliability, with weighted subscale alphas ranging between 0.87 and 0.91. 

The Positive and Negative Affect Schedule (PANAS) is a widely used measure of trait affect. It consists of two 10-item mood scales to measure positive affect (e.g., “excited”, “determined”) and negative affect (e.g., “distressed”, “scared”). Participants rate each item based on a 5-point Likert scale from 1 (very slightly or not at all) to 5 (extremely). It has been shown to have high reliability and construct validity [31]. The negative affect scale showed acceptable reliability within our sample (*a* = 0.75), and the positive affect subscale showed strong reliability within our sample (*a* = 0.91). 

The RAND Health SF-36 Questionnaire (SF-36) was developed as part of the Medical Outcomes Study by RAND Healthcare. It is a measure of health-related quality of life. It comprises two subscales, the Mental Component Score (MCS) as a measure of mental health functioning (e.g., “Have you felt downhearted and blue?”), and the Physical Component Score (PCS) as a measure of physical functioning (e.g., “Does your health now limit you in… climbing several flights of stairs”). There are multiple response scales on the questionnaire, therefore a z-score from 0-100 is calculated for each item, and the items are averaged for each subscale. This scale has been shown to have strong reliability and construct validity in many studies [32]. 

#### 2.2.3. Psychosocial Resource Measures

The Life Orientation Test-Revised (LOT-R) is a 10-item scale that measures one’s general outlook. Three items measure optimism (e.g., “In uncertain times, I usually expect the best”), three items measure pessimism (reverse-scored; e.g., “If something can go wrong for me, it will”), and four items are filler. Participants rate each item on a 5-point Likert scale from 1 (I disagree a lot) to 5 (I agree a lot). The pessimism reverse scored items are averaged with the optimism items to create a total optimism score. It has been shown to have adequate reliability and construct validity [33]. Within our sample, the total scale shows good internal consistency reliability (*a* = 0.85).

The Functional Assessment of Chronic Illness Therapy- Spiritual Wellbeing (FACIT-Sp) measures three dimensions of spiritual wellbeing: meaning (e.g., “I have a reason for living”), peace (e.g., “I feel a sense of harmony within myself”), and faith (e.g., “I find comfort in my faith or spiritual beliefs”). Participants rate each item on a 5-point Likert scale from 0 (not at all) to 4 (very much). Total scores on the 12-item scale range from 0–48. It has been shown to have good internal consistency reliability and good construct validity [34]. This scale has acceptable internal consistency reliability in our sample (*a* = 0.79).

The 12-item Interpersonal Support Evaluation List (ISEL) measures tangible (e.g., “If I were sick, I could easily find someone to help me with my daily chores”), appraisal (e.g., “I feel that there is no one I can share my most private worries and fears with”), and belonging (e.g., “I don’t often get invited to do things with others”) social support. Each item is scored from 0 (definitely false) to 3 (definitely true) and has been found to have good psychometric properties [35]. The ISEL showed excellent internal consistency reliability within our sample (*a* = 0.91). 

### 2.3. Data Analysis 

Following preliminary descriptive analyses, we tested each of our aims using SPSS Statistics Software.

(1) To measure stability, we conducted a paired sample t-test of PTGI to compare means from Time 1 and Time 2. Additionally, we conducted Pearson’s r correlational analysis to determine whether time since diagnosis was associated with PPTG.
(2) To examine how Time 1 PPTG (PTGI, LAYA-SRQL, and IOC) related to our measures of wellbeing, we conducted both longitudinal and prospective Pearson’s correlational analyses. In the first set of analyses, we examined longitudinal correlations between T1 PPTG (PTGI, LAYA-SRQL, and IOC) and T2 levels of wellbeing, encompassing depression, anxiety, and stress (DASS); posttraumatic stress disorder (PTSD) symptoms (IES); concerns about recurrence (CARS); quality of life in the domains of spiritual, social, health, and family (QLI); positive and negative affect (PANAS); and physical and mental health (SF-36).
(3) Next, we conducted prospective analyses by conducting bivariate correlational analyses between our T1 measures of PPTG with change in each wellbeing variable (calculated as T2-T1 for each wellbeing variable).
(4) To examine associations of PPTG with psychosocial resources, we first conducted cross-sectional Pearson’s bivariate correlational analyses between T1 PTGI and three psychosocial resources at T1 (optimism, faith, and social support). Then, we conducted longitudinal Pearson’s bivariate correlational analyses comparing T1 PTGI with T2 psychosocial variables. Finally, we conducted this same analysis but correlated change from T1 to T2 in PTGI with change in psychosocial variables between T1 and T2. Additionally, to provide more specific tests of these associations, we correlated specific subscales of the PTGI with the three psychosocial resources with which they were most closely matched (optimism with PTGI “New Possibilities”, faith with PTGI “Spiritual Change”, and social support with PTGI “Relating to Others”). Each of the aforementioned analyses with psychosocial resources was then conducted using this “matched” approach. To develop our change scores, we subtracted T1 values from T2 values.

## 3. Results

We conducted a two-tailed a priori sensitivity analysis to ensure we would not have false negative or false positive results. With our sample size, we had sufficient power to detect small to medium effect sizes at T1 (r = 0.24; df = 118) and T2 (r = 0.29; df = 86) with 0.80 power at an *a* = 0.05 level.

### 3.1. Attrition Analysis

Based on an independent-samples t-test, completers versus non-completers differed on only two measures. The analysis included demographics, cancer and treatment status, and all measures used in our analyses. Compared to those who dropped out of the study after completing Time 1, those who completed Time 2 were more likely to report that cancer has made them more altruistic/empathetic [IOC altruism/empathy; t = −3.154 (df = 112), *p* < 0.01]. In addition, those who were still in primary treatment were less likely to continue with the study [t = 2.523 (df = 120), *p* < 0.05). 

### 3.2. Stability of PPTG

Participants reported fairly high mean levels of PTGI at both time points [***M***s = 2.98 (***SD***.97) and 2.89 (SD = 1.04), respectively]. Results of a paired *t*-test indicated that Time 1 and Time 2 PTGI did not differ (***t***(86) = 1.07, ***ns***). Furthermore, most participants reported similar levels of PTGI at both time points: PTGI decreased more than 1 point for 8.0% of the sample and increased more than 1 point for 11.6% of the sample. Time since diagnosis was not significantly related to PTGI (***r*** = 0.04, ***ns***).

### 3.3. PPTG and Wellbeing

Results of the longitudinal analyses, shown in Table 2, demonstrated that PTGI was positively correlated with T2 PTSD (IES) but unrelated to other measures of wellbeing. Of the 154 LAYA-SRQL positive scores, only a few were correlated with T2 wellbeing, including PTSD symptomatology, QLI spirituality, family, and health, and positive affect. None of the LAYA-SRQL positive subscales were correlated with fear of recurrence, QLI social, PCS, or MCS. Of the positive scales, only family growth was correlated with the DASS and negative affect. In contrast, of the 154 LAYA-SRQL negative scores, most were correlated with multiple wellbeing indices. In particular, most of the LAYA-SRQL negative scales were correlated with the IES, QLI health, and QLI spiritual subscales, such that declines in survivorship-related quality of life related to poorer wellbeing in general. Among the 4 IOC positive impact subscales, few were significantly correlated with T2 wellbeing, similar to those of the LAYA-SRQL. For the 4 IOC negative impact subscales, almost all were correlated with most of the wellbeing indices with the exception of the PCS, MCS, and positive affect, again in the direction that more negative impact was correlated with lower levels of wellbeing.

In prospective analyses, controlling for Time 1 levels of wellbeing, T1 PTGI was correlated with subsequent declines in positive affect and spiritual QLI, based on wellbeing change scores (T2-T1; see Table 3). Only a few LAYA-SRQL positive change scores and negative change scores were significantly correlated with wellbeing change scores, when controlling for T1 levels of wellbeing. For the T1 IOC positive impact change scores, more significant correlations emerged than in the longitudinal analyses; all of these correlations were in the direction of doing more poorly at Time 2 (e.g., more positive meaning of cancer at T1 was associated with a decline in positive affect by Time 2). For the IOC negative impact subscales, no prospective correlations were statistically significant.

### 3.4. Associations of PPTG with Psychosocial Resources

Overall, T1 PTGI was correlated with two of our three psychosocial resource variables cross-sectionally: faith and social support, as shown in Table 4. The correlation between T1 PTGI and faith remained at T2; however, TI PTGI was not related to changes in any resources as shown in the prospective (resource change) analyses. Change in PTGI was associated with change in faith. 

In analyses examining the PTGI subscales matched to psychosocial resources, T1 PTGI Spiritual Change was strongly correlated with T1 faith and T2 faith, and was correlated with change in faith from T1 to T2. T1 PTGI Relationships was positively correlated with T1 and T2 social support but not with change in social support from T1 to T2. Change in PTGI Relationships was not correlated with change in social support. PTGI new possibilities was not related to optimism at either time point nor to change from T1 to T2. For more details, please see Table 4.

## 4. Discussion

We aimed to test the validity of PPTG as reflecting actual growth in a longitudinal study of adolescent and young adult cancer survivors. Taking three different approaches (stability, relations with wellbeing, and relations with psychosocial resources), our findings overall did not support the validity of PPTG as reflecting actual positive personal change. Rather, our findings are consistent with the handful of prospective studies that have failed to demonstrate validity of PPTG [3] and add to these findings from prospective studies by examining issues of validity over a longer time frame and with a sample dealing with a common traumatic situation.

Based on the stability of perceptions of growth, PTGI scores in our sample appear to represent a more stable, trait-like perception of personal change following cancer. If the PTGI was in fact measuring growth, we would expect to see a continued upward change in PPTG over time. Instead, the results show stable levels of PPTG, suggesting that underlying personal characteristics strongly contribute PPTG. These characteristics may include a general need to believe that things have improved since a traumatic experience, or personality characteristics such as neuroticism, hope, or a belief that everything happens for a reason or is part of a larger plan. However, there may be individual differences in the stability of growth. For example, if reports of growth are serving as efforts to cope with a stressful experience, the need to cope may diminish over time as the situation resolves, reducing the amount of growth perceived. Alternately, PPTG may also strongly inform trauma survivors’ life narratives, the stories that individuals create and maintain about their lives [36], in which case these narratives may remain fixed for the remainder of their lives.

We compared PTGI with a wide range of wellbeing outcomes to give PPTG the most generous chance of showing construct validity. Even still, relationships with wellbeing were sparse. PPTG was only minimally related to wellbeing and in multiple instances, with poorer wellbeing over time. Such findings are consistent with other research demonstrating that PPTG is not associated with psychological adjustment to trauma [24] or to more general wellbeing [12]. While the PTGI was associated with some aspects of wellbeing longitudinally, T1 PTGI was associated with little change in wellbeing over time, further supporting the notion that this scale may be measuring trait-like characteristics rather than accurate reports of actual positive change. 

Importantly, by comparing multiple methods of assessing PPTG, we demonstrated that the valence tapped by the assessment instrument appears to play a role in the relationship between PPTG and wellbeing. Measures that allow individuals to report a range of perceived impact from negative to positive appear to capture important aspects of perceived change that relate fairly consistently with wellbeing. Based on these results, measures with negatively-valenced items or scoring appear to more accurately capture relationships between perceived change and wellbeing than those comprising solely positively-valenced items such as the PTGI. This notion may explain the lack of correspondence that we found between PTGI and wellbeing. The PTGI may be more effective at capturing its intended construct if it were to include either neutrally valenced items, or a full range of positively to negatively valenced items.

The content of the PTGI suggests its intention to measure growth in psychosocial resources. Overall, we did not see strong associations between PTGI and resources longitudinally or with change in psychosocial resources (which would reflect growth rather than a trait), with the exception of spirituality. Such a lack of association does not support the validity of the PTGI as generally reflecting actual change. However, as in previous work [37,38], we found fairly strong associations between PTGI and spirituality, and even between increases in PTGI and increases in spirituality. This finding regarding spirituality suggests that perhaps individuals’ perceptions of their spiritual growth do reflect positive changes in their spirituality, consistent with previous research [3]. However, our matching of PTGI subscales with psychosocial resources was not perfect, and one could argue that “New possibilities” is a poor fit for optimism. On the other hand, our measure of spirituality was specifically a measure of spiritual wellbeing, which is not a perfect fit for spirituality, tapping more of one’s finding comfort in one’s spirituality. Thus, our finding of perceived growth in one’s spirituality and finding more comfort in one’s spirituality may be at least partially due to overlap in the constructs assessed, especially if one is relying on one’s spirituality as a coping resource. 

Limitations of our study must be acknowledged, including the homogeneity of our sample. Our sample was 93% white/Caucasian and largely college-educated (77.2%). Furthermore, more than 60% reported an annual income greater than $60,000. While our findings may be applicable to white, higher socio-economic status populations, these findings may not be generalizable to other populations. Another limitation is the amount of time since diagnosis. At a mean of nearly four years since diagnosis at T1, it is possible that participants are reporting on a different stage of growth, as they are not starting the study at the beginning of survivorship. This timeframe may further cause our reports to be more representative of perceptions of growth than on actual growth and change. However, we did not find a positive correlation between time since diagnosis and amount of PPTG as assessed with the PTGI. Future studies should attempt to conduct these assessments either immediately after diagnosis or treatment completion.

In spite of these limitations, our findings extend our understanding of the validity of PPTG by using several different methods to probe its reflection of actual experiences of positive change. Our findings suggest that PPTG does not tap actual positive changes. Future research should continue to examine the meaning and value of PPTG; narrative approaches may be useful in illuminating how PPTG functions in the long-term stories of individuals’ lives. Meanwhile, new methods should be developed for attempting to better assess true positive changes that individuals experience [9] to further our understanding of resilience and thriving following traumatic experiences.

These findings may have important implications for those working with individuals who have undergone highly stressful experiences, such as life-threatening illnesses or trauma. These individuals may report that they have high levels of personal growth as a result of coping with these stressors, and it is important keep in mind that these perceptions of growth may very well represent something other than veridical reports of positive change. In particular, these reports likely represent individuals’ ongoing efforts to cope with their stressful situations rather than signifying actual instances of positive change. For those conducting counseling or therapy with clients recovering from stressful experiences, it is important to remain aware that these perceptions will likely become key aspects of their life narrative; however, at this point, there is not sufficient evidence of their helpfulness or adaptive value to encourage or promote such perceptions. On the other hand, it seems counter-therapeutic to actively discourage perceptions of growth. Thus, listening to and acknowledging perceptions of PTG, while encouraging clients to be honest in their self-reflection and self-inquiry, seems like a reasonable approach until future evidence on the value of PPTG is reported. More generally, it appears prudent to remain skeptical of self-reported positive changes that one may learn about as related in stories in one’s social networks or via news or social media.

## Figures and Tables

**Table 1:** Sample Demographics.

Demographic	n	%
*Gender*	113	
Female	88	77.9%
Male	25	22.1%
*Race*	114	
Caucasian/White	106	93%
African American/Black	1	0.9%
Asian/Pacific Islander	1	0.9%
Native American	1	0.9%
Other	5	4.3%
*Income/year*	109	
< $20,000	9	8.3%
$20,000-$40,000	16	14.7%
$40,000-$60,000	15	13.8%
$60,000-$80,000	17	15.6%
$80,000-$100,000	19	17.4%
>$100,000	33	30.2%
*Education*	114	
Some High School	1	0.9%
High School Degree	6	5.3%
Some College	19	16.7%
College Degree	56	49.1%
Graduate Degree	32	28.0%
*Cancer Site*	120	
Breast	30	25.0%
Lymphoma	27	22.5%
Thyroid	18	15.0%
Testicular	10	8.3%
Cervix/Uterus/Ovary	7	5.8%
Leukemia/Blood	7	5.8%
Brain	4	3.3%
Colon/Rectal	3	2.5%
Kidney	3	2.5%
Other (melanoma, liver, abdomen, oral)	11	9.3%
*Time Since Diagnosis (year)*	110	
≤1	23	20.9%
2	19	17.3%
3	13	11.8%
4	16	14.5%
5	13	11.8%
>5	26	23.7%
*Time Since Primary Tx ended (years)*	106	
≤1	22	20.8%
2	26	26.5%
3	11	10.4%
4	14	13.2%
5	13	12.2%
>5	20	16.9%
*Treatment Status*	29	
Currently in Primary Treatment	12	9.8%
Evidence of Recurrence	17	14.2%

**Table 2:** Longitudinal Bivariate Correlational Analysis of T1 perceived posttraumatic growth (PPTG) and T2 wellbeing.

	DASS T2	IES T2	FOR T2	QLI Spiritual T2	QLI Social T2	QLI Health T2	QLI Family T2	Positive Affect T2	Negative Affect T2	MCS T2	PCS T2
PTGI Total	0.14	0.23 *	0.12	0.12	0.07	0.03	0.04	0.17	0.14	−0.10	0.04
Intimacy Growth	−0.17	−0.11	−0.20	0.29 *	0.21	0.31 **	0.32 **	0.11	−0.16	−0.06	0.05
Social Growth	−0.02	0.13	−0.02	−0.03	−0.13	−0.10	−0.14	0.13	0.04	0.08	−0.05
Healthcare Growth	−0.08	0.08	−0.03	0.16	0.04	0.14	0.19	0.09	−0.06	0.00	−0.06
Spirituality Growth	0.17	0.25 *	0.12	0.14	0.10	−0.06	0.19	0.05	0.15	−0.04	0.02
Coping Growth	−0.17	−0.03	−0.17	0.34 **	0.07	0.22	0.22	0.38 ***	−0.20	−0.03	−0.12
Dependency Growth	−0.04	0.11	0.01	−0.01	−0.10	0.01	0.13	0.20	−0.06	0.10	−0.19
Cognition Growth	−0.15	−0.04	−0.14	0.17	0.04	0.10	0.17	0.23 *	−0.15	0.01	−0.09
Education Growth	0.01	0.11	−0.06	0.11	0.03	0.05	0.10	0.27 *	0.09	−0.01	−0.05
Fertility Growth	−0.19	−0.13	−0.15	0.28 *	0.24	0.22	0.29 *	0.17	−0.16	−0.07	0.06
Lifestyle Growth	−0.03	0.03	0.08	0.24 *	0.09	0.25 *	0.06	0.51 ***	−0.09	−0.08	−0.10
Romantic Growth	0.11	0.14	−0.04	0.14	0.21	0.06	0.26 *	0.11	0.02	0.02	−0.01
Finance Growth	−0.08	−0.07	−0.16	0.10	0.01	0.13	0.20	0.16	0.29	0.97	0.03
Family Growth	−0.50 ***	−0.33 **	−0.12	0.31 **	0.13	0.38 ***	0.31 **	0.22	−0.44 ***	−0.10	−0.01
Energy Growth	0.20	−0.04	−0.02	0.25 *	0.20	0.26 *	0.16	0.36 **	−0.08	0.01	−0.03
Intimacy Decline	0.35 **	0.39 ***	0.55 ***	−0.24 *	−0.14	−0.41 ***	−0.15	−0.17	0.36 **	0.12	−0.13
Social Decline	0.29 *	0.18	0.19	−0.31 **	−0.20	−0.34 **	−0.22	−0.23 *	0.19	−0.18	0.19
Healthcare Decline	0.31 **	0.31 **	0.11	−0.29 *	−0.38 **	−0.43 ***	−0.33 **	−0.32 **	0.16	0.14	−0.19
Spirituality Decline	−0.02	0.04	−0.04	0.03	0.00	0.05	−0.25 *	−0.06	0.04	0.04	−0.01
Coping Decline	0.45 ***	0.38 ***	0.25 *	−0.42 ***	−0.07	−0.46 ***	−0.18	−0.25 *	0.40 ***	0.06	0.05
Dependency Decline	0.45 ***	0.39 ***	0.07	−0.14	−0.21	−0.36 **	−0.31*	−0.10	0.28 *	−0.09	0.08
Cognition Decline	0.38 **	0.34 **	0.20	−0.29 *	−0.16	−0.34 **	−0.11	−0.33 **	0.29 *	−0.08	0.13
Education Decline	0.44 ***	0.34 **	0.31 **	−0.44 ***	−0.35 ***	−0.56 ***	−0.40 ***	−0.30 *	0.36 **	−0.09	0.13
Fertility Decline	0.17	0.27 *	0.33 **	−0.12	0.02	−0.26 *	−0.12	−0.15	0.24	0.04	0.10
Lifestyle Decline	0.15	0.13	0.15	−0.38 **	−0.31 **	−0.37 **	−0.16	−0.41 ***	0.17	−0.11	0.09
Romantic Decline	0.23	0.25 *	0.42 ***	−0.24 *	−0.18	−0.36 **	−0.42 ***	−0.09	0.24 *	0.05	−0.02
Finance Decline	0.49 ***	0.48 ***	0.30 **	−0.42 ***	−0.45 ***	−0.54 ***	−0.42 ***	−0.31 **	0.40 ***	0.06	0.01
Family Decline	0.56 ***	0.58 ***	0.23 *	−0.27 **	−0.09	−0.50 ***	−0.25	−0.08	0.44 ***	0.08	0.02
Energy Decline	0.34 **	0.25 *	0.15	−0.42 **	−0.32 **	−0.48 ***	−0.14	−0.42 ***	0.33 **	0.06	0.09
IOC Altruism	0.19	0.29 **	0.08	0.09	−0.14	−0.11	−0.20	0.07	0.10	−0.07	−0.04
IOC Health Awareness	−0.14	0.03	0.08	0.26 *	0.14	0.24 *	0.18	0.20	−0.14	0.08	0.04
IOC Meaning of Cancer	0.04	0.16	−0.02	0.18	0.04	0.04	−0.18	0.31 **	−0.01	0.02	−0.06
IOC Positive Eval	−0.11	0.09	−0.01	0.12	0.08	0.13	−0.01	0.26 *	-0.07	−0.06	0.02
IOC Appearance Concern	0.48 **	0.43 ***	0.48 ***	−0.37 **	−0.36 **	−0.44 ***	−0.42 ***	−0.21	0.46 ***	0.08	−0.03
IOC Body Change	0.37 **	0.37 **	0.28 *	0.38 **	−0.32 **	−0.43***	−0.31 **	−0.36 **	0.28 *	0.18	0.02
IOC Life Interfere	0.44 ***	0.58 ***	0.48 ***	−0.30 **	−0.39 ***	−0.52 ***	−0.36 **	−0.23	0.33 **	0.03	0.01
IOC worry	0.46 ***	0.50 ***	0.70 ***	−0.40 ***	−0.34 **	−0.45 ***	−0.28 *	−0.27 *	0.46 ***	0.09	0.02

DASS = Depression Anxiety Stress Scale, IES = Impact of Events Scale; FOR = Fear of Recurrence Scale; QLI = Quality of Life Index; MCS = Mental Component Scale; PCS = Positive Component Scale; PTGI = Posttraumatic Growth Inventory; All items labeled as “growth” or “decline” are pary of the Late Adolescence and Young Adulthood Survivorship Related Quality of Life Scale; IOC = Impact of Cancer Scale. * *p* < 0.05; ** *p* < 0.01; *** *p* < 0.001.

**Table 3:** Bivariate Correlational Analysis of T1 PPTG with to T2-T1 change in Wellbeing.

	DASS Change	IES Change	FOR Change	QLI Spiritual Change	QLI Social Change	QLI Health Change	QLI Family Change	Positive Affect Change	Negative Affect Change	MCS Change	PCS Change
PTGI Total	0.10	0.17	−0.01	−0.25 *	−0.09	−0.12	−0.14	−0.23 *	−0.05	0.04	0.03
Intimacy Growth	0.00	−0.04	−0.07	0.12	−0.05	0.02	0.10	0.03	0.05	−0.11	0.06
Social Growth	−0.04	0.10	0.01	−0.24 *	−0.38 **	−0.21	−0.20	−0.19	−0.06	0.04	−0.06
Healthcare Growth	0.14	0.06	0.06	−0.19	−0.33 **	−0.26 *	−0.04	−0.19	0.12	0.10	0.05
Spirituality Growth	0.13	0.18	−0.03	−0.22	−0.04	−0.08	−0.12	−0.21	0.07	0.01	0.01
Coping Growth	0.02	0.09	0.12	−0.25 *	−0.21	−0.38 ***	−0.06	−0.13	0.25 *	0.07	0.06
Dependency Growth	0.08	0.13	−0.09	−0.25 *	−0.29 *	−0.24 *	0.01	0.00	−0.02	0.20	−0.08
Cognition Growth	−0.11	0.06	−0.07	−0.09	−0.16	−0.19	−0.03	0.01	−0.03	0.04	−0.03
Education Growth	0.03	0.05	0.05	−0.15	−0.24 *	−0.20	0.04	−0.07	0.12	−0.14	0.04
Fertility Growth	0.36	0.29	0.33	0.29*	0.14	0.11	0.19	0.03	−0.01	−0.22	0.10
Lifestyle Growth	−0.03	0.05	0.08	−0.09	−0.06	−0.18	−0.01	0.01	−0.01	0.04	−0.04
Romantic Growth	0.23	0.10	−0.22	0.00	0.09	−0.09	−0.04	0.02	−0.11	0.11	−0.01
Finance Growth	0.03	−0.12	−0.18	−0.05	−0.12	−0.10	−0.03	−0.08	−0.05	−0.13	0.00
Family Growth	−0.17	−0.15	0.05	0.10	−0.34 **	0.12	0.07	0.14	0.08	0.09	−0.19
Energy Growth	−0.01	−0.02	0.05	−0.04	−0.10	−0.07	−0.03	−0.04	0.05	0.07	−0.11
Intimacy Decline	−0.08	−0.09	−0.13	0.16	0.18	0.09	−0.03	0.12	−0.06	0.10	−0.08
Social Decline	−0.16	−0.11	−0.17	0.11	0.32 **	0.17	0.17	0.22	−0.30 *	−0.06	0.24 *
Healthcare Decline	0.06	0.12	−0.05	−0.17	0.06	−0.02	−0.16	−0.18	−0.04	−0.03	−0.19
Spirituality Decline	−0.10	−0.01	0.17	0.08	−0.08	0.05	0.10	0.01	−0.05	0.09	−0.16
Coping Decline	0.01	−0.04	−0.25 *	0.09	0.29 *	0.19	0.03	0.15	−0.32 **	0.07	0.03
Dependency Decline	0.07	0.12	−0.01	0.03	0.14	0.04	0.02	0.07	−0.16	−0.08	0.11
Cognition Decline	0.05	−0.13	−0.10	0.08	0.21	0.09	−0.04	0.14	−0.07	−0.14	0.19
Education Decline	0.06	0.08	−0.02	−0.13	0.28 *	0.01	−0.09	0.02	−0.25 *	−0.03	0.09
Fertility Decline	−0.01	0.01	−0.07	−0.12	0.07	−0.03	−0.07	−0.08	−0.04	0.06	0.14
Lifestyle Decline	−0.01	0.00	−0.10	−0.02	0.15	0.03	0.10	0.18	−0.01	0.00	0.14
Romantic Decline	−0.27 *	−0.13	0.07	0.21	0.18	0.03	−0.03	0.12	−0.17	0.04	0.02
Finance Decline	0.10	0.13	−0.10	−0.16	0.12	−0.06	−0.08	−0.08	−0.19	0.12	−0.02
Family Decline	0.21	0.36	−0.02	−0.07	0.37 **	−0.18	−0.05	0.07	−0.16	−0.01	0.13
Energy Decline	−0.04	−0.11	−0.19	0.00	0.18	0.03	0.14	0.22	−0.10	−0.02	0.26 *
IOC altruism	−0.01	0.16	−0.14	−0.02	−0.13	−0.11	−0.04	−0.01	−0.07	0.00	0.05
IOC Health Awareness	−0.18	−0.08	−0.13	−0.03	−0.17	0.07	−0.04	−0.18	0.00	0.12	0.00
IOC Meaning of Cancer	0.12	0.24 *	0.13	−0.33 **	−0.22	−0.35 **	−0.28 *	−0.31 **	0.18	0.05	0.00
IOC Positive Evaluation	−0.01	0.18	0.01	−0.28 *	−0.29 *	−0.23 *	−0.25 *	−0.27 *	0.11	0.14	−0.11
IOC Appearance Concern	0.10	0.22	0.04	−0.08	0.08	−0.04	−0.22	−0.05	0.11	0.09	0.12
IOC Body Change	−0.08	−0.05	−0.12	0.01	0.12	0.12	−0.05	−0.04	−0.16	0.06	0.12
IOC Life Interference	−0.07	0.13	−0.06	0.06	0.09	0.01	0.07	0.10	−0.22	0.07	0.16
IOC worry	0.11	0.07	−0.16	0.02	0.08	0.04	−0.11	−0.03	−0.13	0.11	0.00

DASS = Depression Anxiety Stress Scale, IES = Impact of Events Scale; FOR = Fear of Recurrence Scale; QLI = Quality of Life Index; MCS = Mental Component Scale; PCS = Positive Component Scale; PTGI = Posttraumatic Growth Inventory; All items labeled as “growth” or “decline” are pary of the Late Adolescence and Young Adulthood Survivorship Related Quality of Life Scale; IOC = Impact of Cancer Scale. * *p* < 0.05; ** *p* < 0.01; *** *p* < 0.001.

**Table 4:** Bivariate Correlational Analyses of T1 Post Traumatic Growth Inventory (PTGI) and PTGI corresponding subscales with psychosocial resources (Cross-sectional and change).

	PTG T1	Change in PTG T2-T1	PTG T1 Corresponding Subscale	PTG T2 Corresponding Subscale	Change in PTG T2-T1 Corresponding Subscale
T1 Optimism LOT-R	0.10	0.02	0.09	0.12	0.00
T1 Faith FACIT-Sp	0.44 **	−0.25 *	0.76 ***	0.67 ***	−0.19
T1 Social Support ISEL	0.21 *	−0.16	0.38 ***	0.19	−0.24 *
T2 Optimism	0.19	−0.03	0.16	0.09	−0.07
T2 Faith	0.47 ***	−0.06	0.70 ***	0.74 ***	0.07
T2 Social Support	0.17	−0.07	0.27 *	0.08	−0.15
Change in Optimism T2-T1	0.17	−0.18	0.10	−0.01	−0.15
Change in Faith T2-T1	−0.07	0.26 *	−0.16	0.07	0.37 **
Change in Social Support T2-T1	−0.15	0.12	−0.10	−0.01	0.09

PTG = Posttraumatic Growth Inventory; LOT-R = Life Orientation Test-Revised; FACIT-Sp = Functional Assessment of Chronic Illness Therapy- Spiritual Wellbeing; ISEL = Interpersonal Support Evaluation List; * *p* < 0.05; ** *p* < 0.01; *** *p* < 0.001.
